# KLF4α stimulates breast cancer cell proliferation by acting as a KLF4 antagonist

**DOI:** 10.18632/oncotarget.10058

**Published:** 2016-06-15

**Authors:** Jacqueline Ferralli, Ruth Chiquet-Ehrismann, Martin Degen

**Affiliations:** ^1^ Friedrich Miescher Institute for Biomedical Research, Novartis Research Foundation, Basel, Switzerland; ^2^ Faculty of Science, University of Basel, Basel, Switzerland; ^3^ Department of Orthodontics and Dentofacial Orthopedics, School of Dental Medicine, University of Bern, Bern, Switzerland

**Keywords:** alternative splicing, KLF4, KLF4α/KLF4(FL) ratio, proliferation, tumors

## Abstract

Krüppel-like factor 4 (KLF4), a transcription factor involved in both tumor suppression and oncogenesis in various human tumors, is subject to alternative splicing that produces KLF4α. *KLF4α* is primarily expressed in the cytoplasm because it lacks exon 3 of *KLF4*, which contains the nuclear localization signal. The role of KLF4 in breast cancer remains unclear and nothing is known yet about the expression and function of the isoform KLF4α. Here, we show that *KLF4α* is expressed in normal and tumoral tissue of the breast and provide evidence that the KLF4α/KLF4(full-length) (FL) ratio is increased in tumors compared to corresponding normal tissue. Forced increase of the KLF4α/KLF4(FL) ratio in the metastatic breast cancer cell line MDA-MB-231 decreases the levels of *E-Cadherin, p21^Cip1^*, and *p27^Kip1^*, three known KLF4(FL) target genes, and stimulates cell proliferation. We suggest that cytoplasmic KLF4α binds to KLF4(FL) and retains it in the cytoplasm thereby antagonizing the gene regulatory activities of KLF4(FL) in the nucleus. Our results establish KLF4α as a KLF4 isoform that opposes the function of KLF4(FL) and as an important factor in the complex and unresolved role of KLF4(FL) in breast carcinogenesis.

## INTRODUCTION

Krüppel-like factors (KLFs) comprise a highly conserved transcription factor family of 17 members [1]. They are known to regulate cellular functions such as proliferation, apoptosis, migration, differentiation, and pluripotency [2–4]. KLF4 is the best-studied member of the family and it is one of the factors in the “Yamanaka cocktail”, which upon transfection, allows the conversion of adult cells into induced pluripotent stem cells [5].

KLF4 has been extensively studied in the context of tumors and current data suggest that it can either act as tissue-specific tumor-inhibiting or -promoting gene with the underlying mechanism remaining unclear [6, 7]. KLF4 has been reported to have tumor-suppressive functions in various tumors, including tumors of the colon, bladder, prostate, and stomach [8–13], while it acts as a pro-tumorigenic gene in oral and skin squamous cell carcinomas [14, 15].

The role of KLF4 in breast cancer remains less clear and contradictory data exist. It is believed that KLF4 is expressed at low levels in normal breast epithelium, but over-expressed during breast tumor progression [16]. Furthermore, increased nuclear KLF4 expression is considered to be a marker of an aggressive phenotype in early-stage infiltrating ductal carcinoma [17]. Additionally, KLF4 plays a prominent role in the maintenance of the cancer stem cell-like population, which promotes cell migration and invasion [18]. However, the role of KLF4 as a putative tumor-promoting gene in breast cancer remains unresolved: extensive analyses of the Oncomine database revealed lower KLF4 mRNA levels in breast tumor tissues, compared with normal tissues, in 9 of 11 data sets and indicated that the levels are inversely correlated with tumor grade [19]. Moreover, two new genetic variants in the estrogen-receptor positive breast cancer susceptibility locus 9q31.2 [20] have been recently identified that target KLF4 via long-range chromatin interactions [21]. These data show that lower levels of KLF4 are associated with increased breast cancer risk and promote KLF4 as a tumor suppressor-like gene [20, 21]. Similarly, KLF4 inhibits epithelial-mesenchymal-transition (EMT) and metastasis in breast cancer models [22–24]. Thus, KLF4's role in breast tumors remains a conundrum.

Only recently, KLF4 isoforms have been identified [25–27]. One of the main KLF4 isoforms, KLF4α, has been shown to be over-expressed in pancreatic cancers and to correlate with the aggressiveness of tumors and poor patient prognosis [26].

Here, we aimed to elucidate whether KLF4α is expressed in breast cancer cells and, if so, whether increased KLF4α levels might explain some of the complexity of KLF4's role in breast tumorigenesis. We have established that KLF4α is expressed in the cytoplasm of breast cancer cells and present evidence that an increased *KLF4α/KLF4(FL)* ratio is often found in tumors compared to normal tissue. Our data suggest that KLF4α acts as a dominant KLF4(FL) antagonist and prevents nuclear translocation of KLF4(FL), thereby altering the transcriptional landscape in breast cancer cells. We provide evidence that KLF4α has tumor-promoting functions and that its expression may play a significant role in KLF4's complex functions in breast cancer.

## RESULTS

### Detection of *KLF4α* in human breast cancer cells

**Figure 1:**
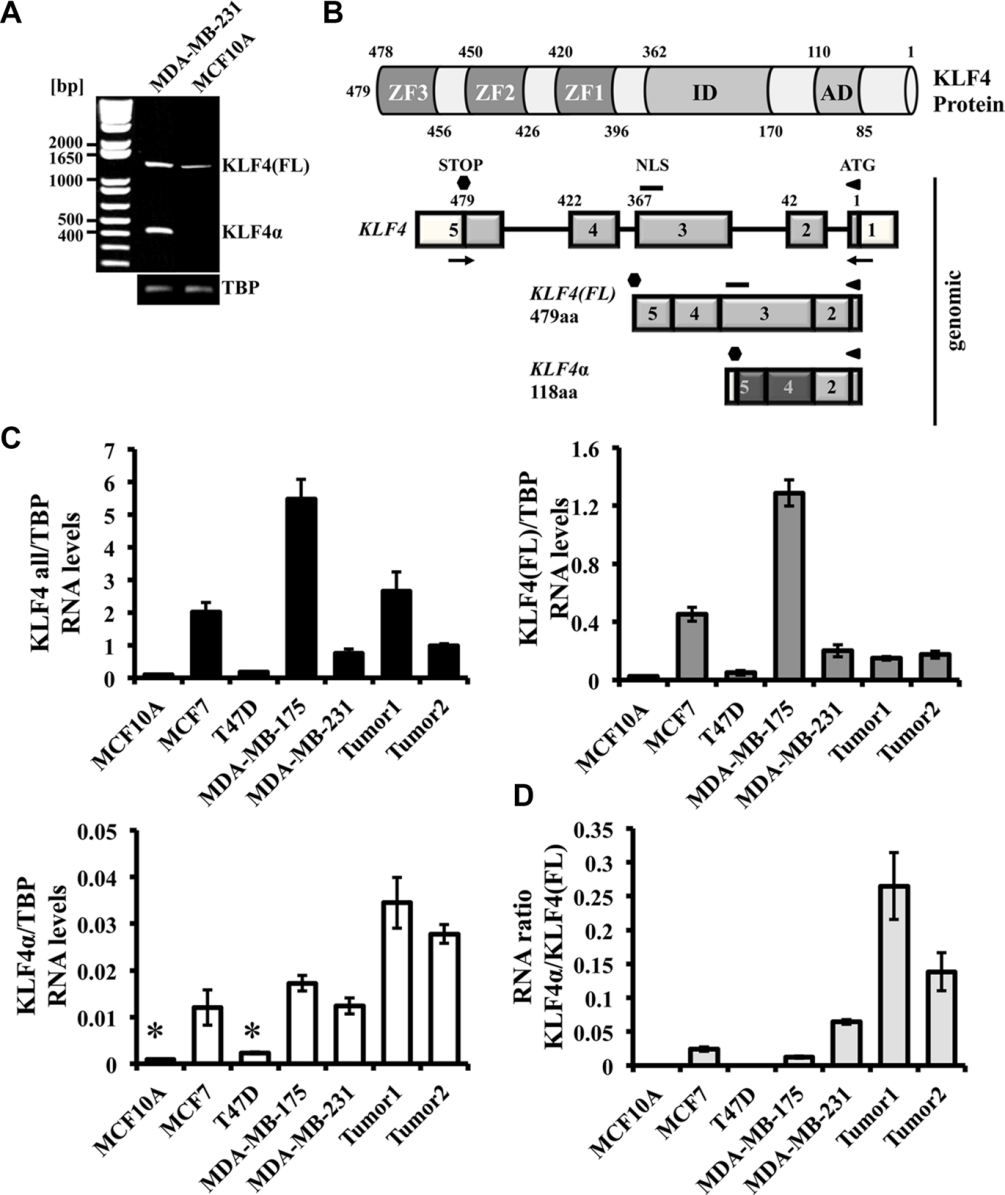
Detection of KLF4α in human breast cancer cells (**A**) RT-PCR analysis of *KLF4* in MDA-MB-231 and MCF10A cells. Both cell lines show the KLF4(FL) band (∼1440 bp), whereas the KLF4α band (∼440 bp) is only detectable in MDA-MB-231 cells. (**B**) Schematic representation of the KLF4 protein structure (top) and the *KLF4* gene and its variants in MDA-MB-231 cells (bottom). The *KLF4* gene is located on chromosome 9 (reverse strand), contains 5 exons, which gives rise to a KLF4(FL) protein of 479aa. Skipping of exon3 produces KLF4α (118aa). Numbers indicate the amino acids. AD: activation domain; ID: inhibitory domain, ZF: zinc fingers; NLS: nuclear localization signal; aa: amino acids; white boxes: untranslated exons; light grey boxes: shared sequence between KLF4(FL) and KLFα; dark grey boxes: novel sequence in KLF4α; arrows: primers used in A). (**C**) qPCR analysis of a normal human breast cell line (MCF10A) compared to four human breast cancer cell lines and two human ductal breast carcinoma patients for the genes *KLF4 all*, *KLF4(FL)*, and *KLF4α*. Note that *KLF4α* is detectable in three out of the four breast cancer cell lines and that the two breast cancer patient samples show high levels of *KLF4α* compared to cells. Data are expressed as the mean +/− SEM. *n* = 3. TBP: TATA-Box binding protein. *C_T_ values ≥ 34. (**D**) RNA ratio of *KLF4α/KLF4(FL)* indicates highest ratio in the two cancer patient samples.

Unresolved data on the role of KLF4 during breast carcinogenesis [4], as well as the identification of KLF4α, a KLF4 isoform, as a tumor-promoting gene in pancreatic cancer [26], prompted us to study KLF4α expression in breast cancer cells. To test whether normal and/or breast cancer cells express *KLF4*α, we used MCF10A and MDA-MB-231 cells and performed RT-PCR with primers flanking the *KLF4* gene (Figure 1A, 1B). A product of ∼1440 bp was amplified in both cell lines, while a ∼440 bp amplicon was detectable in the metastatic MDA-MB-231 cells only (Figure 1A). Sequencing of these PCR products revealed *KLF4(FL)* (1440 bp band; UniProtKB-O43474; KLF4 isoform 2) and *KLF4α* (440 bp; UniProtKB-O43474-5). *KLF4α* is a *KLF4* isoform that lacks exon3, leading to a frameshift in exon4 and to a premature Stop codon in exon5 (Figure 1B and Supplementary Figure S1). All three zinc finger domains of KLF4(FL) and its nuclear localization signal (NLS) are not present in KLF4α.

Next, we wanted to quantitatively study RNA levels of the two *KLF4* variants in a panel of breast cancer cell lines (MCF7, T47D, MDA-MB-175, and MDA-MB-231), the normal human breast cell line (MCF10A), and also in samples from patients with ductal carcinoma. Specificity of qPCR primers recognizing *KLF4(FL)* and *KLF4α*, respectively, as well as primers that detect both *KLF4* variants (“KLF4 all”) (Supplementary Figure S2) allowed us to study breast cancer-associated *KLF4* splicing in more detail. *KLF4 all* levels were variable in all our samples analyzed (Figure 1C, left). *KLF4(FL)* levels mostly paralleled those of *KLF4 all*, with the exception of the carcinoma patient 1 (Figure 1C, right). In three out of the four breast cancer cell lines tested, *KLF4α* RNA was readily detectable (Figure 1C bottom). Only T47D cells were negative for *KLF4α*. *KLF4α* was not detectable in the normal breast cell line MCF10A, which confirmed our RT-PCR (Figure 1A). The relative expression patterns of *KLF4(FL)* and *KLF4α* in the cell lines were very similar. In the two patient samples, however, *KLF4*α showed higher expression than in cell lines, which was in contrast to *KLF4(FL)*. Since both KLF4 variants were detectable in most of the breast cancer samples, we wished to determine the *KLF4α/KLF4(FL)* ratio in each sample. *KLF4α/KLF4(FL)* ratios were variable across the samples, but highest in the carcinoma patients, which was due to their elevated *KLF4α* (Figure 1D).

So far, there is only limited data on KLF4α expression in cancer cell lines [25, 26]. Thus, we decided to screen an additional panel of 21 human cancer cell lines from various origins for the expression of *KLF4 all*, *KLF4(FL),* and *KLF4α* (Supplementary Figure S3). This analysis demonstrated that *KLF4α* transcripts are expressed in 84% of the cancer cell lines tested, (including breast cancer; Supplementary Table 1).

### *KLFα* in human tumors

**Figure 2:**
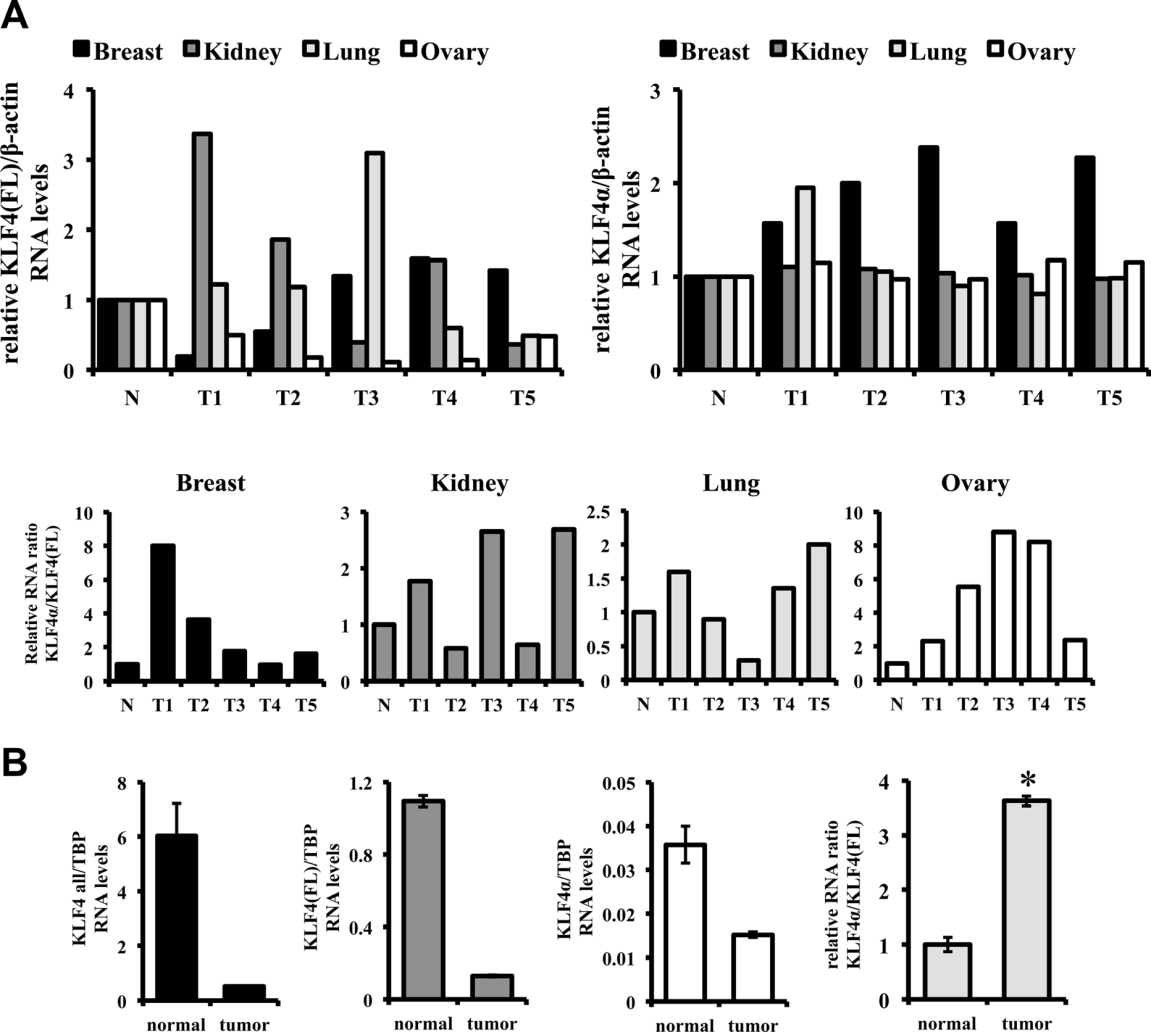
*KLF4α/KLF4(FL)* imbalance in tumors (**A**) qPCR analysis for *KLF4(FL)* and *KLF4α* in TissueScans containing 5 different tumor samples (T1-5) and a normal control tissue (N). Consistent increases of *KLF4α/KLF4(FL)* are detected in 4/5 breast, 3/5 kidney, 3/5 lung, and 5/5 ovary cancer samples compared to controls. (**B**) A matched pair of a ductal carcinoma breast patient was analyzed by qPCR for *KLF4(FL)* and *KLF4α*. While *KLF4all*, *KLF4(FL)* and *KLF4α* RNA levels are decreased in tumors, there is a prominent *KLF4α/KLF4(FL)* ratio imbalance in the tumor sample compared to the control tissue. TBP: TATA-Box binding protein. Data are expressed as the mean +/− SEM. *n* = 3. *p ≤ 0.05 (tumor versus normal).

To extend this study and to analyze clinically relevant specimens, we used a TissueScan^TM^ Cancer and Normal Tissue cDNA Array. This array consists of five breast, kidney, lung, and ovary cancer samples and one normal control for each tissue (Supplementary Figure S4). qPCR analysis showed that *KLF4α* transcripts were detectable in all control tissues (Figure 2A, right). *KLF4α* expression was two-fold higher in normal kidney, lung, and ovarian tissue compared to normal breast (data not shown). *KLF4α* RNA was also prominently expressed in all the different tumor patients. Comparing the levels of *KLF4α* in control and tumor samples, no consistent difference could be observed in kidney, lung, and ovarian tumor patients. Only in the five breast cancer patients *KLF4α* was consistently and prominently over-expressed compared to control tissue (Figure 2A right). *KLF4(FL)* was detectable in all normal tissues as well (Figure 2A, left) with highest expression in ovarian tissue and lowest levels in breast tissue (data not shown). In ovarian tumors all patients displayed a prominent reduction of *KLF4(FL)* levels confirming literature on tumor-suppressive functions of KLF4 in ovarian cancer [28]. When we determined the ratio *KLF4α/KLF4(FL)* in all these clinical samples, we noticed an appreciable increase of the ratio in 4/5 breast, 3/5 kidney, 3/5 lung, and 5/5 ovary cancer samples compared to their corresponding healthy tissues (Figure 2A bottom panels).

To further solidify our hypothesis of an increased *KLF4α/KLF4(FL)* ratio in tumors, we used a matched pair RNA sample from an invasive ductal carcinoma and adjacent normal tissue (Figure 2B). *KLF4 all*, *KLF4(FL)* as well as *KLF4α* RNA levels were all prominently reduced in the tumor sample compared to control. Still, a significant increase of the *KLF4α/KLF4(FL)* ratio in the tumor could be determined (Figure 2B).

### Cytoplasmic localization of KLF4α

**Figure 3:**
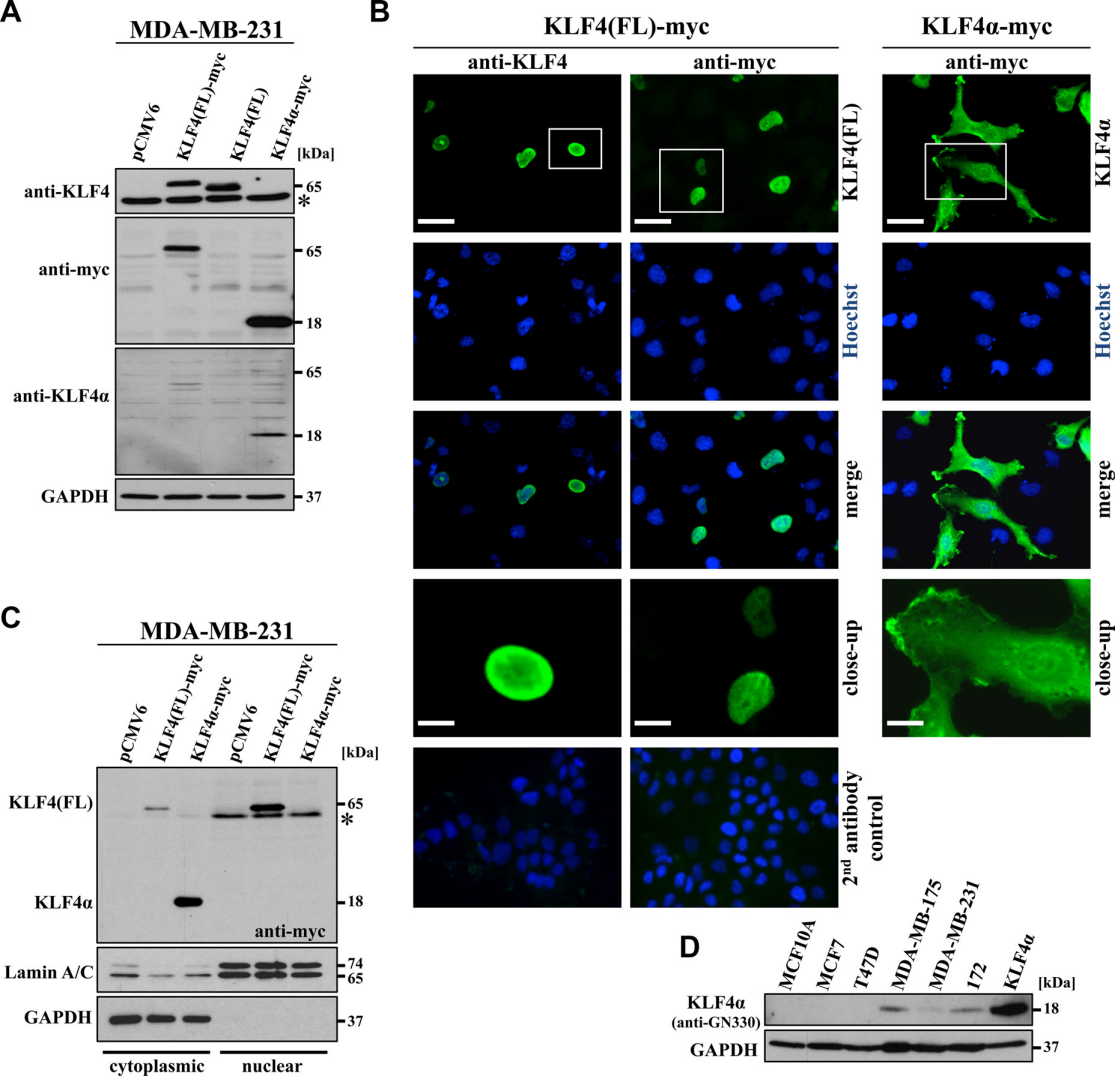
Characterization of KLF4α expression (**A**) MDA-MB-231 cells were transfected with the indicated plasmids and immunoblotted with α-KLF4, which only detects KLF4(FL), with α-myc, which detects both myc-tagged variants of KLF4, and with α-GN330, which specifically recognizes KLF4α. *background band. (**B**) Immunofluorescent stainings of transfected MDA-MB-231 cells 24 h post-transfection show nuclear staining for KLF4(FL) and cytoplasmic staining for KLF4α. Hoechst stain was used for the visualization of nuclei. Scale bar: 50 μm (top); 10 μm (close-up). The bottom row indicates the secondary antibody only controls merged with the respective Hoechst staining (left side: goat-anti-rabbit IgG-FITC; right side: goat-anti-mouse IgG-FITC). (**C**) Transfected MDA-MB-231 cells were extracted for the cytoplasmic and the nuclear fractions 36 hours after transfection and immunoblotted. KLF4α is only found in the cytoplasmic, GAPDH-positive fraction, whereas most of KLF4(FL) is present in the nuclear, LaminA/C-positive fraction. * background band. (**D**) Western blot analysis of protein extracts of the normal breast cell line (MCF10A), four breast cancer cell lines as well as a p53-mutant Li Fraumeni cell line (172) in comparison to KLF4α-transfected MDA-MB-231 cells for KLF4α levels. Note that endogenous KLF4α (∼18kDa) is detectable in MDA-MB-175, MDA-MB-231, and 172 cells. GAPDH: glyceraldehyde-3-phosphate dehydrogenase.

To study KLF4(FL) and KLF4α function in breast cancer cells, we cloned both cDNAs into mammalian expression plasmids and transfected MDA-MB-231 cells. The KLF4 antibody used in this study was raised against a C-terminal peptide corresponding to human KLF4 aa 300–400 and therefore, only detected KLF4(FL) (∼65 kDa). KLF4α (∼18 kDa) could be detected by its myc-tag as well as by the antibody anti-GN330 [26] (Figure 3A). *KLF4(FL)* contains a NLS in exon 3 (Figure 1B), which is responsible for its nuclear localization (Figure 3B). In contrast, KLF4α, lacking exon3, was localized primarily in the cytoplasm of MDA-MB-231 cells (Figure 3B, right panel). Complementary localization of KLF4(FL) and KLF4α was further confirmed by preparing cytoplasmic and nuclear protein extracts of transfected MDA-MB-231 cells (Figure 3C). KLF4α expression was only detected in the cytoplasmic fraction, while the vast majority of KLF4(FL) was present in the nuclear fraction (Figure 3C), which confirmed our staining results.

Specificity of the anti-GN330 antibody for KLF4α (Figure 3A) allowed us to analyze endogenous KLF4α protein levels in our breast cancer cells. KLF4α was detected in extracts of MDA-MB-175, MDA-MB-231, and 172 (mutated p53 showing high KLF4α RNA levels (Supplementary Figure S3C)), but not in T47D and MCF10A cells (Figure 3D). These results confirmed our RNA analyses (Figure 1C), suggesting a good correlation between KLF4α RNA and protein levels.

### Effects of KLF4(FL) and KLF4α in MDA-MB-231 cells

**Figure 4:**
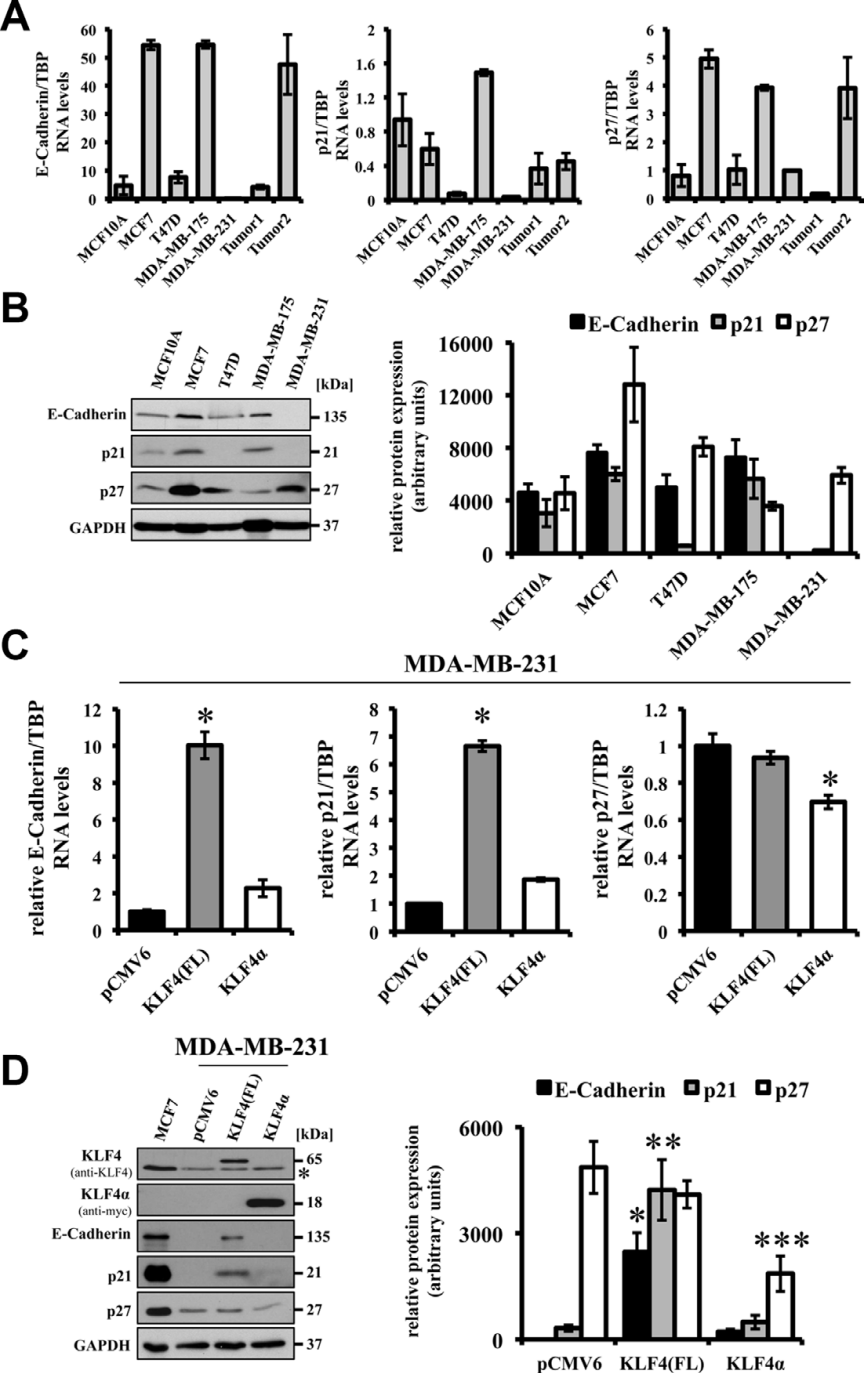
Effects of forced expression of KLF4(FL) and KLF4α in MDA-MB-231 cells (**A**) qPCR analysis of a normal human breast cell line (MCF10A) compared to 4 human breast cancer cell lines and two human ductal breast carcinoma patients for endogenous levels of *E-Cadherin*, *p21^Cip1^* and, *p27^Kip1^* shows various expression levels in the different samples. Note that *E-Cadherin* and *p21^Cip1^* levels are very low in MDA-MB-231. Data are expressed as the mean +/− SEM. *n* = 3. (**B**) Western Blot analysis of the normal and cancerous breast cells for E-Cadherin, *p21^Cip1^*, *p27^Kip1^* indicates that protein levels correlate with RNA levels. Normalized protein levels of three independent western blots have been quantified and plotted. (**C**) Forced expression of KLF4(FL) in MDA-MB-231 cells induces *E-Cadherin* as well as *p21^Cip1^* RNA, but not *p27^Kip1^*. In contrast, forced expression of KLF4α does neither affect basal levels of *E-Cadherin* nor *p21^Cip1^*, but decreases *p27^Kip1^*. Data are expressed as the mean +/− SEM. *n* = 3. TBP: TATA-Box binding protein. *p ≤ 0.05 (KLF4(FL) versus pCMV6/KLF4α). (**D**) Response of E-Cadherin, *p21^Cip1^*, and *p27^Kip1^* protein levels upon forced expression of KLF4(FL) and KLF4α, respectively, in MDA-MB-231 cells in comparison to the endogenous protein levels in MCF7. Normalized protein levels of three independent western blots have been quantified and plotted. *p ≤ 0.05 E-Cadherin in KLF4(FL) versus KLF4α cells; **p ≤ 0.05 *p21^Cip1^* in KLF4(FL) versus KLF4α cells; ***p ≤ 0.05 *p27^Kip1^* in KLF4α versus KLF4(FL) cells. Note that KLF4α antagonizes KLF4(FL)-mediated target gene regulation and decrease *p27^Kip1^*. GAPDH, glyceraldehyde-3-phosphate dehydrogenase. *background band.

Reported tumor-suppressive effects of forced KLF4 expression include the induction of the epithelial cell-adhesion molecule E-Cadherin and the cell cycle inhibitors p21^Cip1^ and p27^Kip1^ via direct binding of KLF4 to the respective promoter elements [22, 29, 30]. In our panel of breast cancer cells we detected strong variation of endogenous E-Cadherin, p21^Cip1^, and p27^Kip1^ RNA and protein levels. High *E-Cadherin*, *p21^Cip1^*, and *p27^Kip1^* levels mostly correlated with high expression of *KLF4(FL)* in the breast cancer cell lines (Figures 1C, 4A). MDA-MB-231 cells, which are believed to have undergone an EMT, hardly express any E-Cadherin and p21^Cip1^ (Figure 4A, 4B).

Forced expression of KLF4(FL) in the highly metastatic MDA-MB-231 cells robustly restored E-Cadherin RNA as well as protein expression (Figure 4C left panel, D lane 3). Similarly, KLF4(FL) over-expression induced p21^Cip1^, while it was not able to stimulate p27^Kip1^ expression in MDA-MB-231 cells (Figure 4C right panel, 4D). These results suggest that KLF4(FL) appears to enforce an epithelial phenotype and to induce growth arrest by increasing E-Cadherin and p21^Cip1^ levels, respectively. Indeed, KLF4(FL) over-expressing MDA-MB-231 cells display a more cobblestone-like, epithelial morphology and have growth disadvantages compared to control cells (data not shown). In contrast, an increased KLF4α/KLF4(FL) ratio by forced KLF4α expression in MDA-MB-231cells was not able to either induce E-Cadherin or p21^Cip1^, but it decreased p27^Kip1^ levels compared to control (Figure 4C, 4D). Comparable results were obtained in KLF4(FL) and KLF4α over-expressing MCF7 cells (data not shown)

### KLF4α antagonizes KLF4(FL) function

**Figure 5:**
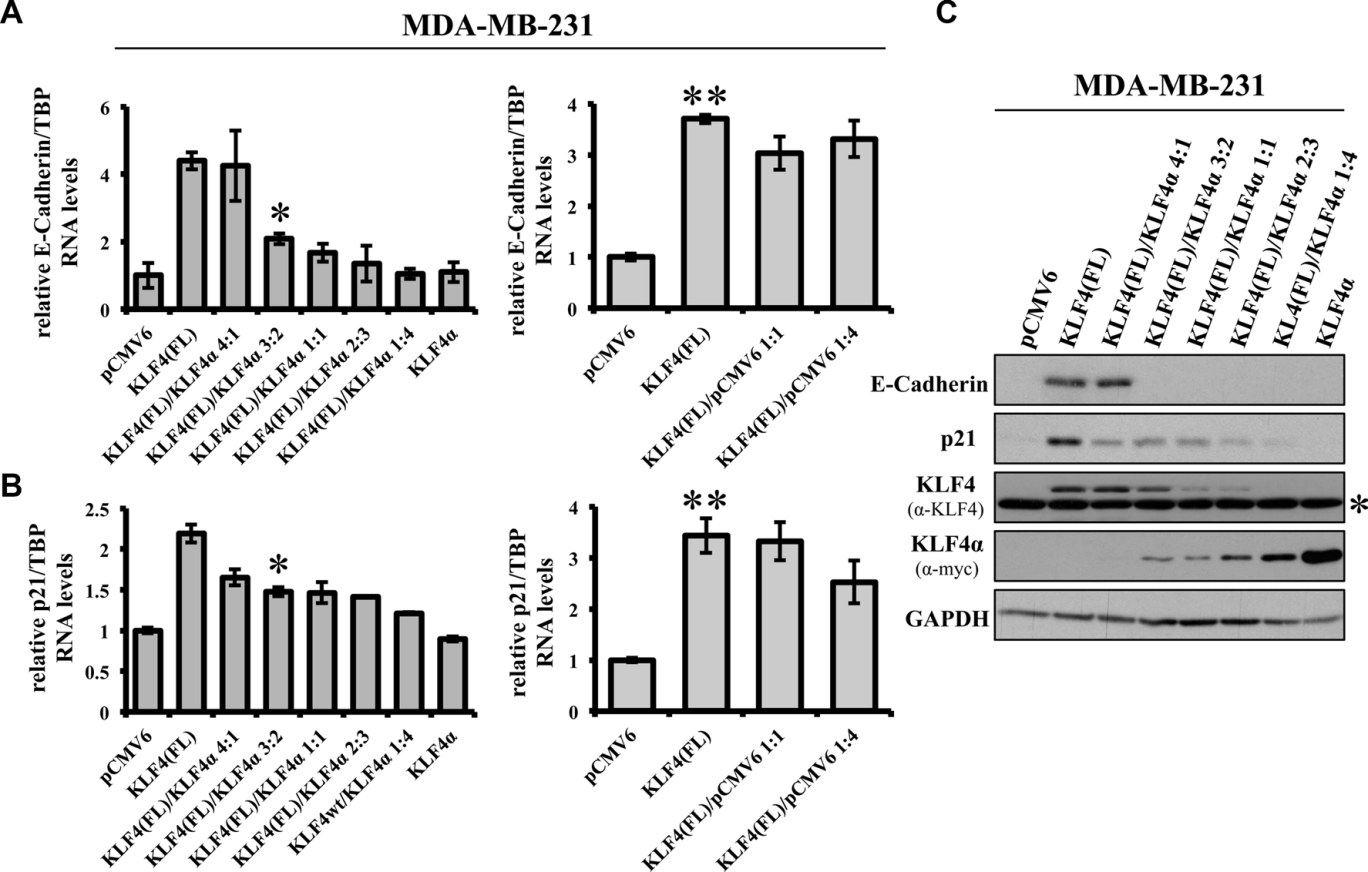
KLF4α antagonizes KLF4(FL)-mediated effects on E-Cadherin and p21 (**A**) MDA-MB-231 cells were transiently transfected with different ratios of KLF4α and KLF4(FL) followed by qPCR analysis for *E-Cadherin*. Increasing amounts of KLF4α block KLF4(FL)-mediated *E-Cadherin* RNA induction (left panel). As a control, different ratios KLF4(FL)/pCMV6 were used followed by qPCR for *E-Cadherin* levels. Note that even KLF4(FL)/pCMV6 ratio of 1:4 still robustly induced *E-Cadherin* (right panel). Data are expressed as the mean +/− SEM. *n* = 3. *p ≤ 0.05 (KLF4(FL) versus (FL)/α 3:2). **p ≤ 0.05 (KLF4(FL) versus pCMV6). (**B**) KLF4α/KLF4(FL) imbalance in MDA-MB-231 cells reduces KLF4(FL)-provoked *p21^Cip1^* induction as evidenced by qPCR (left panel). Right panel shows the control using different KLF4(FL)/pCMV6 ratios. Data are expressed as the mean +/− SEM. *n* = 3. TBP: TATA-Box binding protein. *p ≤ 0.05 (KLF4(FL) versus (FL)/α 3:2). **p ≤ 0.05 (KLF4(FL) versus pCMV6). (**C**) MDA-MB-231 cells were transiently transfected with different ratios of KLF4α and KLF4(FL) followed by immunoblot analysis using specific antibodies for E-Cadherin and p21^Cip1^. Note that KLF4α/KLF4(FL) imbalances are reflected on protein levels as assessed by anti-KLF4 (KLF4(FL)) and anti-myc (KLF4α) and abrogate E-Cadherin and p21^Cip1^ inductions. GAPDH, glyceraldehyde-3-phosphate dehydrogenase. *background band.

Next, we wanted to know whether an increased KLF4α/KLF4(FL) ratio, which we had identified in tumors, might have consequences for KLF4(FL) target genes. Therefore, we generated KLF4α/KLF4(FL) imbalances and analyzed their effects on KLF4's ability to induce E-Cadherin and p21^Cip1^ in MDA-MB-231 cells. Co-transfection of different amounts of KLF4(FL) and KLF4α, respectively, altered their individual protein levels in a dose-dependent manner, thereby changing the KLF4α/KLF4(FL) ratios (Supplementary Figure S5 and Figure 5C). Increased KLF4α/KLF4(FL) ratios abrogated KLF4(FL)-mediated induction of E-Cadherin and p21^Cip1^ (Figure 5A, 5B left panels, 5C). In contrast, similar ratios of KLF4(FL)/pCMV6 ratios were still capable of inducing both genes (Figure 5A, 5B right panels). Our data demonstrate that increased KLF4α/KLF4(FL) ratios, either generated by reduction of KLF4(FL) or gain of KLF4α, antagonizes KLF4(FL)-mediated induction of E-Cadherin and p21^Cip1^.

### KLF4α interacts with KLF4(FL)

**Figure 6:**
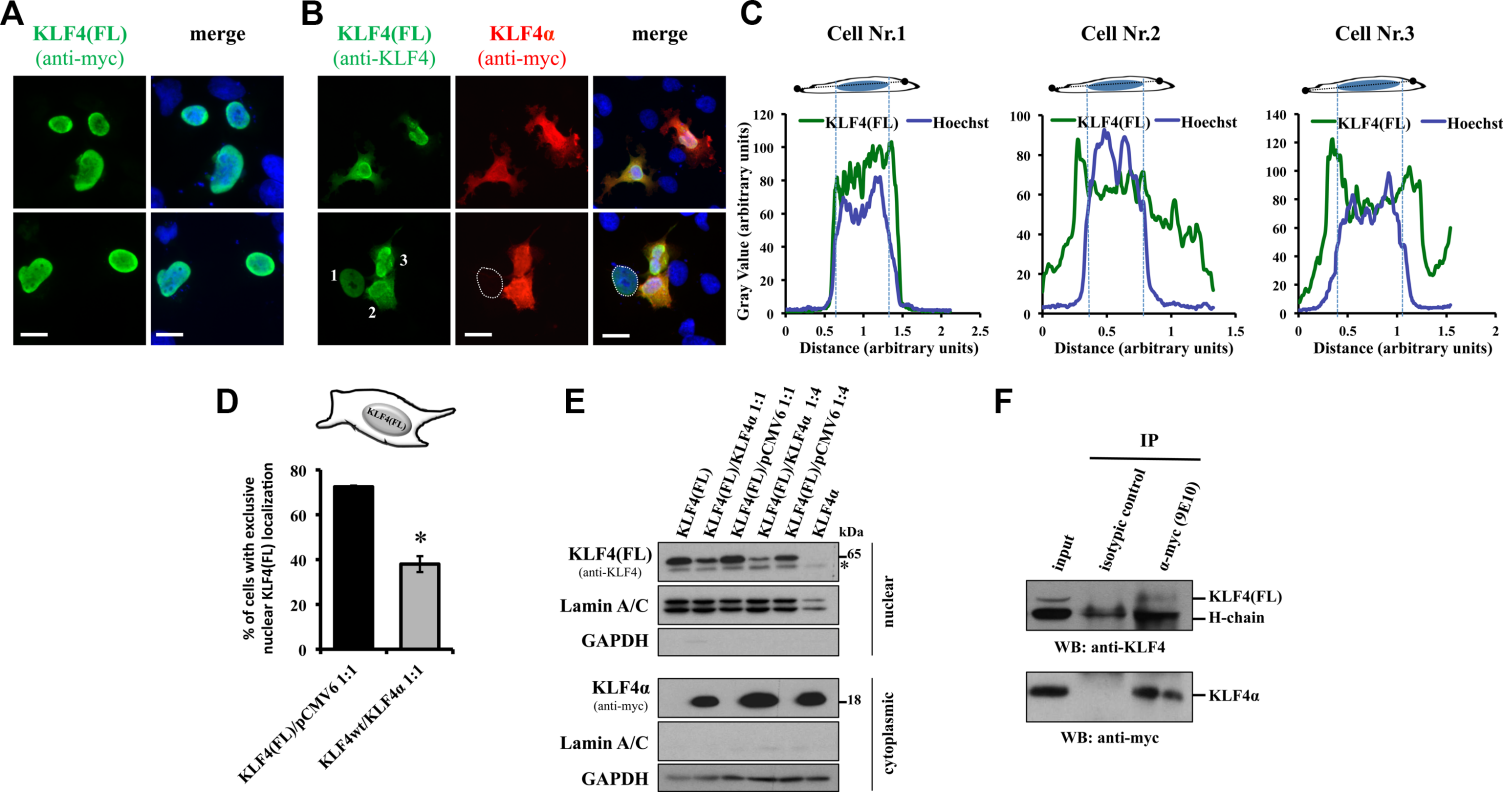
KLF4α sequesters KLF4(FL) in the cytoplasm (**A**) MDA-MB-231 cells co-transfected with a 1:1 mix of KLF4(FL)-myc and pCMV6, and stained 24 h after transfection. Two sample images are shown. KLF4(FL)-myc is mostly located in the nucleus overlapping with the Hoechst stain. scale bar: 20 μm. (**B**) MDA-MB-231 cells transfected with a 1:1 mix of KLF4(FL) and KLF4α-myc, and stained 24 h after transfection. Two sample images are shown. In KLF4α-positive cells, KLF4(FL) is often localized in the cytoplasm. In the bottom row, the dotted line indicates a KLF4α-negative cell that displays an exclusive nuclear staining for KLF4(FL). Hoechst staining to indicate the nuclei is shown in blue. scale bar: 20 μm. (**C**) Linescan plots of the three cells in panel B, bottom are shown. Note, while in the absence of KLF4α the linescan plots for KLF4(FL) (green line) and Hoechst (blue line) are almost identical (indicating strict nuclear KLF4(FL) expression in cell 1), presence of KLF4α alters KLF4(FL) localization (cell 2 and 3). (**D**) Quantification of the co-transfection experiments. 100 KLF4α-positive cells were analyzed for the exclusive nuclear localization of KLF4(FL) (*n* = 3). Graph shows difference of percentage of cells having strict nuclear KLF4(FL) expression in the KLF4(FL)/pCMV6 versus the KLF4(FL)/KLF4α co-transfected cells. *p ≤ 0.05 (KLF4(FL)/pCMV6 versus KLF4(FL)/KLF4α). (**E**) Nuclear extracts of MDA-MB-231 cells co-transfected with different ratios of KLF4α/KLF4(FL) analyzed by immunoblots. Note that altering the KLF4α/KLF4(FL) ratio decreases nuclear KLF4(FL) levels compared to the respective ratios of KLF4(FL)/pCMV6. Nuclear extracts were defined by the presence of LaminA/C and the absence of GAPDH. GAPDH, glyceraldehyde-3-phosphate dehydrogenase. *background band. (**F**) Co-immunoprecipation analysis of the association between KLF4α and KLF4(FL) using total cell extracts of MDA-MB-231 cells transiently transfected with a 1:1 ratio KLF4α-myc/KLF4(FL) 36 h after transfection. Total cell extract (input) was used as control in western blots. Immunoprecipitation with α-myc followed by western blot with α-KLF4 detects a specific band at 65kDa, which corresponds to KLF4(FL). Bottom panel shows that anti-myc (9E10) immunoprecipitated KLF4α-myc successfully.

An antagonistic function of KLF4α on KLF4(FL) suggests that there might be an interaction of the two proteins. We hypothesized that KLF4α binds KLF4(FL) in the cytoplasm, thereby preventing its translocation into the nucleus. To study this possibility, we co-transfected MDA-MB-231 cells with a 1:1 ratio of either KLF4α-myc/KLF4(FL) or KLF4(FL)-myc/pCMV6 and analyzed the cellular localization of KLF4(FL) by immunofluorescence (Figure 6A, 6B). KLF4(FL) was primarily localized in the nucleus in KLF4(FL)/pCMV6 transfected cells (Figure 6A). In contrast, presence of KLF4α significantly disturbed the nuclear localization of KLF4(FL), as evidenced by cytoplasmic staining patterns (Figure 6B). Typical examples are shown in Figure 6B. In the bottom row of Figure 6B, one out of three cells shows an exclusive nuclear staining (indicated by the dotted line) for KLF4(FL). This cell does not express KLF4α, whereas the two other cells that express KLF4α, have KLF4(FL) in the cytoplasm as well. These observations were confirmed by quantifying the three cells of Figure 6B bottom row by linescan plots (Figure 6C). We further quantified these findings in more than 100 transfected, KLF4α-positive MDA-MB-231 cells. We determined a statistically significant difference in the percentage of cells with exclusively nuclear KLF4(FL) in KLF4(FL)/pCMV6 versus KLF4(FL)/KLF4α transfected cells (Figure 6D). These data let us speculate that KLF4α sequesters KLF4(FL) in the cytoplasm and consequently, KLF4(FL) levels in the nucleus should be decreased in the presence of KLF4α. To test this hypothesis, we co-transfected MDA-MB-231 cells with different KLF4α/KLF4(FL) ratios and analyzed their nuclear fractions for the levels of KLF4(FL). A prominent decrease of nuclear KLF4(FL) was observed in the presence of KLF4α compared to control (pCMV6) (Figure 6E). Finally, we assessed the possibility of a direct or indirect interaction between KLF4α and KLF4(FL) in the cytoplasm of MDA-MB-231 cells by co-IP experiments. Indeed, KLF4α and KLF4(FL) associate with each other as evidenced by the appearance of a KLF4(FL) band in the KLF4α-myc-immunoprecipitated sample (Figure 6F).

### KLF4α stimulates breast cancer cell proliferation

**Figure 7:**
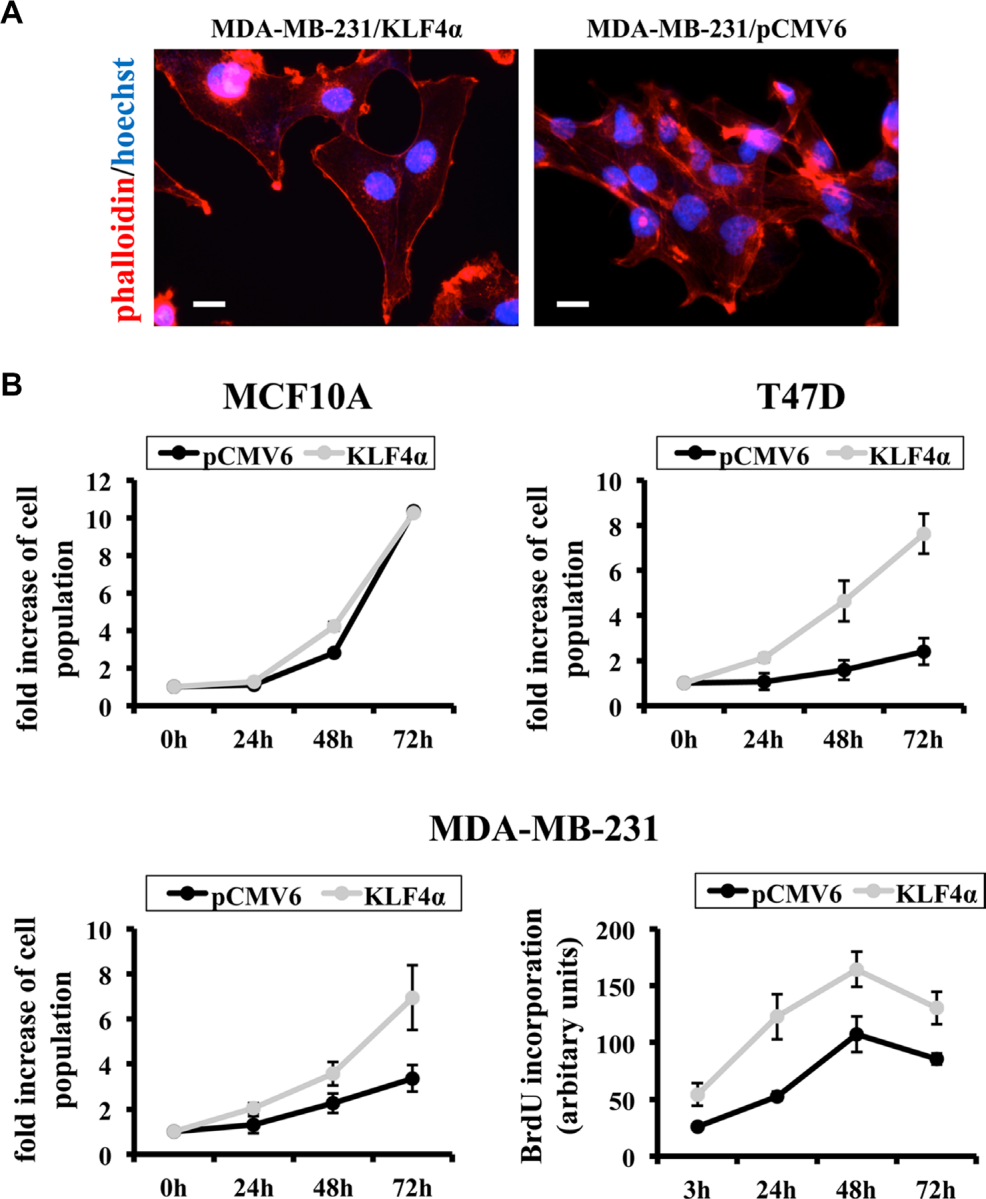
KLF4α stimulates proliferation in MDA-MB-231 cells (**A**) Forced over-expression of KLF4α in MDA-MB-231 cells results in changes of the actin network as evidenced by phalloidin (red). Note the prominent cortical actin in KLF4α expressing cells as compared to control. (**B**) While forced expression in the normal breast cell line MCF10A does not alter cell growth, it does stimulate proliferation in the breast cancer cell lines T47D and MDA-MB-231. Two different assays were used: Top and bottom left panels show the result of counting the cells after the indicated times. The bottom right panel displays BrdU incorporation. *p ≤ 0.05 (KLF4α versus pCMV6).

Finally, we wanted to learn more about the functional consequences of increased KLF4α/KLF4(FL) ratios in MDA-MB-231 cells. First, we analyzed the actin cytoskeleton of KLF4α-over-expressing MDA-MB-231cells compared to control. While the control cells showed high actin expression throughout the cells, we noticed prominent cortical actin and mostly absence of intracellular actin in the KLF4α-cells, which also seemed to be larger than controls (Figure 7A). Since distinct actin network patterns might reflect changes in cellular motility, we wanted to check whether KLF4α stimulates cancer cell migration. Boyden chamber assays as well as scratch wounds were performed and analyzed, but no significant difference in the migratory behavior of KLF4α over-expressing MDA-MB-231 cells compared to control was observed (data not shown). Finally, we tested the role of altered KLF4α/KLF4(FL) ratios on cell growth. While forced KLF4α expression in the normal mammary cell line MCF10A did not change proliferation, the growth rate of T47D and MDA-MB-231 was increased upon KLF4α over-expression (Figure 7B). Hence, KLF4α only had a growth-promoting effect on breast cancer cell lines, which was independent of their respective endogenous total KLF4 levels (low in T47D, high in MDA-MB-231) (Figure 1C). BrdU incorporation assays further demonstrated that increased KLF4α/KLF4(FL) ratios by forced KLF4α expression increased the fraction of cells within S phase (Figure 7B, bottom right). Thus, we propose KLF4α as a novel, so far neglected oncogenic factor, which clarifies the role of KLF4 in breast cancer.

## DISCUSSION

It is well established that KLF4 has ambiguous roles in human breast cancer. The general discovery of KLF4 variants [25–27] and our data on KLF4α as an antagonist of KLF4(FL) in breast cancer might shed new light on the role of KLF4 during breast tumorigenesis.

In the current study, we analyzed the expression and function of the main KLF4 isoform, KLF4α, in breast cancer cells. We demonstrate that *KLF4α* is expressed in breast cancer cells and show that KLF4α is primarily localized in the cytoplasm. Strong evidence is presented that KLF4α sequesters KLF4(FL) in the cytoplasm through an association of KLF4α with KLF4(FL). Consequently, direct or indirect binding of cytoplasmic KLF4α to KLF4(FL) prevents KLF4(FL) from nuclear translocation and results in altered transcriptional regulation of KLF4(FL) target genes, such as *E-Cadherin*, *p21^Cip1^*, and *p27^Kip1^*. Chromatin IP's have shown physical interaction of KLF4(FL) with the promoter of all three genes and KLF4-binding sites have been identified suggesting that KLF4(FL) transactivates E-Cadherin, p21^Cip1^, and p27^Kip1^ [22, 29, 30]. We confirmed the ability of KLF4 to induce levels of E-Cadherin and p21^Cip1^ in the highly metastatic breast cancer cell line MDA-MB-231, but did not observe an increase of p27^Kip1^ upon forced KLF4 expression. However, increasing the KLF4α/KLF4(FL) ratio in MDA-MB-231 strongly antagonized the effect of KLF4(FL) and decreased levels of E-Cadherin, p21^Cip1^, as well as p27^Kip1^. Augmented KLF4α/KLF4(FL) ratios increased the fraction of cells within S phase and promoted a pro-tumorigenic phenotype by opposing the tumor-suppressive functions of KLF4(FL). Since we detected *KLF4α* in human cancers of various origin, we speculate that our findings in breast cancer may be relevant for other tumors as well.

An antagonistic effect of KLF4α on KLF4(FL) function can be explained by two potential mechanisms: Either there is an interaction of KLF4α with KLF4(FL) in the cytoplasm, which then retains KLF4(FL) in the cytoplasm and inhibits nuclear translocation of KLF4(FL), or KLF4α sequesters important co-factors in the cytoplasm required for KLF4(FL)-mediated gene regulation in the nucleus. We cannot exclude the latter, but we showed that KLF4α is associated with KLF4(FL) in the cytoplasm and that nuclear KLF4(FL) levels are decreased in the presence of KLF4α compared to control cells. There is no evidence in the literature that KLF4 can form dimers. Therefore, the detailed molecular mechanism how KLF4α associates with KLF4(FL) remains to be elucidated. It is plausible to speculate that alternative splicing produces novel motifs in KLF4α that are responsible for the interaction or association with KLF4(FL). However, we did not identify any obvious structural motifs in KLF4α.

Similar to our study on KLF4, it has been shown that splicing deregulation of KLF6 results in a specific variant, KLF6-v1, that is associated with an increased risk of various cancers and opposes KLF6(FL) effects [31–37]. It is believed that intronic and/or exonic site mutations and splicing factor alterations are the main reasons for aberrant splicing to occur in tumors [38]. We do not have any data on mutations affecting KLF4 splicing or splicing factor level alterations in human tumors. In the case of KLF6, it has been shown that a single germline DNA polymorphism is responsible for the generation of KLF6-v1 in prostatic tumors [35]. To our knowledge, nothing is known yet about polymorphisms within crucial splice-site regions for the *KLF4* gene in tumors. Further studies are needed to address the important questions of KLF4 splicing deregulation in tumors.

KLF4α expression was initially identified in pancreatic cancer [26]. In pancreatic cancer patients, high KLF4α levels were associated with an aggressive tumor phenotype and poor survival. Pancreatic tumor models showed that KLF4α over-expression resulted in larger tumors, due to increased cancer cell proliferation [26]. Our data on KLF4α in breast cancer mostly confirms this study with some significant novel additions and relevant differences: (i) Compared to Wei et al. [26] our sequencing analysis showed that MDA-MB-231 cells contain a KLF4 protein with nine additional N-terminal amino acids (MRQPPGESD) corresponding to the EMBL Nucleotide Sequence Database accession number HF546201. This N-terminal variance in KLF4 has recently been identified and characterized for the potential of reprogramming [39]. (ii) KLF4α expression has been described in pancreatic and prostatic cancer only [25, 26]. We extended this expression analysis by screening a total of 25 human cancer cells from 11 different tissues and identified *KLF4α* transcripts in 21 out of the 25 cell lines tested (84%). These results indicate that *KLF4α* is widely expressed in human tumors and that our findings in breast cancer may have relevance in other tumors as well. (iii) While KLF4α was not expressed in normal pancreatic tissue [26], we clearly established *KLF4*α expression in normal tissues. In our limited set of normal human tissues, *KLF4α* was readily detectable in all of them (breast, kidney, lung, and ovary). (iv) KLF4α expression was absent in normal pancreas, but correlated with tumor grade in pancreatic cancer patients [26]. We did not identify higher KLF4α levels in kidney, lung, and ovarian cancer patients compared to their respective controls. A tendency towards increased *KLF4α* levels compared to control was only observed in breast cancer patients. It is evident that larger sample numbers will be required to further clarify this point. However, we believe that not KLF4α levels *per se* are important for its actions, but rather that the KLF4α/KLF4(FL) ratio determines whether KLF4α can antagonize KLF4(FL) or whether KLF4(FL) can mediate its transcriptional gene regulation. Most of the tumor samples analyzed in this study displayed a significant increase of the *KLF4α/KLF4(FL)* ratio compared to controls. Tumors can achieve this imbalance either by increased expression of KLF4α, or by reduced levels of KLF4(FL). Numerous studies report a loss of KLF4(FL) expression in tumors (e.g., [3]), suggesting that KLF4(FL) prevents tumor formation. However, it is reasonable to hypothesize that not the loss of KLF4(FL) *per se* is tumorigenic, but that a potential resulting increase of KLF4α/KLF4(FL) ratio might stimulate tumor growth. Therefore, future studies should analyze KLF4(FL) as well as KLF4α levels in cancer patients. (v) We used a cellular model which allowed us to specifically manipulate the KLF4α/KLF4(FL) ratio and to follow the effects of altered KLF4α/KLF4(FL) ratios on E-Cadherin and p21^*Cip1*^. In transfected MDA-MB-231 cells, a KLF4α/KLF4(FL) mix of 1:1 was sufficient to inhibit KLF4(FL)-mediated regulation of the transcriptional targets E-Cadherin and p21^Cip1^. Importantly, these artificially generated KLF4α/KLF4(FL) ratios are in the range of the ratios observed in tumor patients.

In summary, this is the first study on *KLF4*α expression in breast cancer. We show that *KLF4α* is expressed in various normal tissues, but that tumors display an increase of the KLF4α/KLF4(FL) ratio compared to control. We demonstrate that although KLF4(FL) acts as a tumor-inhibiting gene in MDA-MB-231 cells, an increased KLF4α/KLF4(FL) ratio is able to oppose this effect. KLF4α interacts with KLF4(FL) in the cytoplasm, which prevents nuclear localization of KLF4(FL), thereby inhibiting KLF4(FL) tumor-suppressive functions in the nucleus, such as growth inhibition. These results warrant further studies on the role of KLF4α in tumorigenesis using larger sets of clinical samples. Future studies should be aimed at analyzing whether targeted inhibition of KLF4α suppresses breast cancer cell growth. Also, it will be interesting to see how KLF4α/KLF4(FL) imbalance affects the cancer stem cell-like population, which plays an important role in tumor development and emergence of therapy-resistant clones in breast cancer. Finally, it has to be analyzed whether presence of KLF4α, a KLF4 antagonist, might be an important, so far neglected player in the generation of induced pluripotent stem cells.

## MATERIALS AND METHODS

### Cell culture, transfection, stable cells

MDA-MB-231 were purchased from the American Cell Type Culture collection, and cultured according to the manufacturer's instructions under standard conditions. Supplementary Table S1 lists all cell lines used in this study.

For transfections, cells were split the day before so that they reached 60-70% confluence. Transfections were performed using JetPei (Polyplus, Illkirch-Graffenstaden, France) following their protocol with 1 μg DNA and 2 μl JetPei reagent per 35 mm dish. 5 h after transfection, the transfection mix was removed and cells were replenished with fresh medium. Cells were analyzed 24 - 48 h post-transfection.

For the generation of stable MDA-MB-231 cells, expression plasmids (backbone vector: pCMV6-A-Puro, Origene, Rockville, USA) containing a puromycin selection marker were used for transfection. 24 h after transfection, puromycin-selection was started (2 μg/ml; Sigma-Aldrich, St. Louis, USA). Every other day, fresh medium including puromycin was added and selective pressure maintained until the emergence of resistant cell clones. Stable cells were characterized by quantitative real-time PCR (qPCR), immunofluorescence, and immunoblotting.

For the over-expression experiments, pools of cells have been used and experiments have been repeated at least three times after independent transfections.

### Cloning of the KLF4 constructs

cDNA was synthesized from total RNA extracted from MCF10A and MDA-MB-231 cells. PCR was performed on the cDNA's using Taq Polymerase (Roche, Rotkreuz, Switzerland) and the following primers: 5′-ATGAGGCAGCCACCTGGCGAG-3′/5′- CATCGGAGCGGGCGAATTTCC-3′. Amplicons were separated on agarose gels, purified using the QIAquick Gel Extraction kit (QIAGEN, Hilden, Germany) and then used for sequencing.

To generate a myc-tagged KLF4(FL) expression plasmid, the following primers were used: 5′-TGCGATC GCCATGAGGCAGCCACCTGGCGAGTCTG-3′/5′- ATA CGCGTAAAATGCCTCTTCATGTGTAAGGC-3′. The primer set 5′-ATGCGATCGCCATGAGGCAGCCACCT GGCGAGTCTG-3′/5′-ATACGCGTGTTCATCTGAGC GGGCGAATTTC-3′ was used for cloning a myc-tagged KLF4α expression construct. Primers contained an *AsiSI* (forward primer) and a *MluI* restriction sites (reverse primer), respectively. This allowed the directional cloning into the mammalian expression plasmid pCMV6-A-Puro (Origene) containing a myc tag. All expression plasmids were sequence-verified.

### RNA extraction, cDNA synthesis, qPCR analysis

Total RNA was isolated from approximately 80% confluent cells using the RNeasy Mini Kit (QIAGEN). Total RNA was further purified by the Turbo DNAse Treatment and Removal kit (ThermoFisher Scientific, Lucerne, Switzerland). cDNA was synthesized from 1 μg total RNA using the High Capacity cDNA Reverse Transcription kit (ThermoFisher Scientific). mRNA levels were quantified by qPCR using Platinum SYBR Green qPCR SuperMix-UDG with ROX (Invitrogen) on an ABI StepONE Plus Instrument (40 cycles of 95°C for 15 s and 58°C for 30 s). Relative expression was calculated using the ΔΔC^T^ method, normalizing values to TATA-Box binding protein (TBP) within each sample; standard error of the mean (SEM) was calculated from the results of triplicates. All primers were tested for specificity and efficiency. Primers used are listed in Supplementary Table S2.

TissueScan Real-Time Arrays were obtained from Origene. qPCR analysis of the TissueScans was performed according to their manual.

Total RNA from human breast tissues (ductal carcinoma) and a matched pair RNA sample from breast tissue were purchased from Amsbio (Abingdon, UK).

### Western blotting

Cell extracts were prepared in RIPA buffer as described [40]. Alternatively, cultures were rinsed with phosphate-buffered saline (PBS), drained, and lysed in reducing Laemmli sample buffer. After boiling the samples for 5 min at 95°C, proteins in Laemmli buffer were separated by SDS-PAGE under reducing conditions and blotted to polyvinyl-difluoride membranes (ThermoFisher Scientific). Then, membranes were stained with amido black to control for equal protein loading and blotting efficiency. After blocking for 1 h at room-temperature in Tris-buffered saline (TBS) containing 0.05% Tween and 5% skim milk powder (Sigma-Aldrich), membranes were incubated over-night with primary antibodies at 4°C. Membranes were washed three times in TBS-Tween and incubated for 1 h with peroxidase-conjugated anti-rabbit/mouse IgG at room temperature. Blots were developed using SuperSignal West Dura (ThermoFisher Scientific) and exposed to Super RX Fuji Medical X-Ray films (Fujifilm, Diesldorf, Switzerland).

Primary antibodies used: anti-myc (clone 9E10), anti-KLF4 (ab151733) and anti-GAPDH (ab9485, all from Abcam, Cambridge, UK), anti-LaminA/C (#612162, BD Biosciences, East Rutherford, USA), anti-E-Cadherin (#3195), anti-p21^Cip1^ (#2947), and anti-p27^Kip1^ (#3686, all from Cell Signaling Technologies, Danvers, USA). The KLF4α-specific antibody GN330 [26] was a generous gift from Profs. Keping Xie and Daoyan Wei (MD Anderson Cancer Center, University of Texas, USA).

Some Western Blots were analyzed densitometrically using ImageJ software version 1.51a (NIH, Bethesda, MD; http://rsbweb.nih.gov/ij). Briefly, the total band intensity of the protein of interest was normalized to the GAPDH band intensity of the same extract in the same experiment.

### Cytoplasmic and nuclear protein extraction

For preparation of cytoplasmic and nuclear cell extracts, cells grown in 10 cm dishes were washed in ice-cold PBS and lysed in lysis buffer (10 mM HEPES pH 7.9, 100 mM KCl, 1 mM EDTA, 1 mM DTT, 0.5% NP-40 and protease inhibitor cocktail (Roche)). After centrifugation, supernatants were collected as cytoplasmic fractions. Nuclear pellets were washed with lysis buffer lacking NP-40, and extracted with 250 mM Tris-HCl pH 7.8, 100 mM KCl, 1 mM EDTA, 1 M DTT, 0.5% NP-40, 20% glycerol shaking at 4°C for 1 h, followed by centrifugation to clarify the nuclear extracts. Proper fractionation was confirmed by GAPDH and LaminA/C presence as cytoplasmic and nuclear marker, respectively.

### Co-immunoprecipitation

For co-immunoprecipitations (co-IP), cells grown in 10 cm dishes were lysed in ice-cold co-IP buffer (50 mM Tris-HCl, pH 7.4, 1% Triton-X-100, 25 mM Hepes, 150 mM NaCl, 0.2% Sodium deoxycholate, 5 mM MgCl^2^ and protease inhibitor cocktail Roche). Cell lysates were incubated for 60 min on a rotary wheel at 4°C. After centrifugation for 15 min at 13200 rpm, the supernatant was transferred into a new 1.5 ml tube and protein concentration measured by a Bradford Assay (Expedeon, Swavesey, UK). Equal amounts of cell lysates (200 μg) were pre-cleared for 1 h at 4°C on a rotary wheel. Pre-cleared lysates were incubated with 3 μg of anti-myc (9E10) antibody or 3 μg of control mouse IgG (mAB002, R&D Systems, Minneapolis, USA) and incubated over-night at 4°C on a rotary wheel. Dynabeads Protein G (Life Technologies) were added to the immunocomplexes and incubated for 1 h at 4°C on a rotary wheel. After 3 washes with co-IP buffer, complexes were eluted in denaturing SDS sample buffer, resolved by SDS-PAGE and analyzed by immunoblotting

### Immunofluorescence and quantification

For stainings, cells were grown in 35 mm dishes containing four separate wells (Greiner Bio-One, Frickenhausen, Germany). Cells were washed twice with PBS before fixation in 4% paraformaldehyde at room-temperature. Afterwards, cells were washed three times with PBS, permeabilized in 0.1% Triton-X-100 for 5 min and incubated with primary antibody for 2 h at room-temperature. After three PBS rinses, cells were incubated with fluorescent-labeled secondary antibodies (Molecular Probes) for 1 h in the dark, rinsed with PBS and coverslip-mounted with ProLong Gold antifade reagent (Molecular Probes). Hoechst was added to the last washing step. Cells were examined and photographed using an Axioskop microscope (Carl Zeiss MicroImaging, Oberkochen, Germany) connected to an ORCA-ER digital camera (Hamamatsu, Solothurn, Switzerland).

Linescan plots were performed using ImageJ software version 1.51a. Briefly, a line, spanning an entire cell, was drawn and the gray intensities along the line (through the cell) were plotted.

For quantification, 100 cells per experimental group were analyzed in triplicates. KLF4(FL) in KLF4α-positive cells was categorized as “strictly nuclear” or “nuclear+cytoplasmic”. Quantification was blinded and performed by two lab members.

### Cell growth

Cell growth was analyzed by two different methods. First, 9 × 10^4^ cells were plated into 35 mm dishes in 1% FCS-containing medium. At the indicated time points, cells were trypsinized and counted. The increase in cell population was calculated as fold induction compared to the time-point 0. Second, the fraction of cells within S phase was determined using a 5-bromo-2′-deoxyuridine (BrdU) incorporation assay (Roche) as described in their manual. Briefly, 5 × 10^3^ cells were plated into black 96-well plates with clear, flat bottom (PerkinElmer, Waltham, USA) in 1% FCS-containing medium. Cells were allowed to proliferate for the indicated times before labeling with BrdU for 2 h at 37°C. Afterwards, cells were fixed for 30 min, and incubated with the anti-BrdU antibody conjugated with peroxidase for 90 min, followed by extensive washing. BrdU substrate was added and incubated for 3 min before measuring chemiluminescence signals using a Mithras LB940 luminometer (Berthold Technologies, Zug, Switzerland).

### Statistical analysis

Data are represented as means and standard deviation/standard error of the mean (SD/SEM) as stated in the figure legends. Statistical analysis using a two-tailed *t*-test was carried out either at www.physics.csbsju.edu/stats/*t*-test.html or with the program GraphPadPrism Version 5. All experiments have been performed at least 3 times in triplicates. The difference between two data sets was statistically significant when *p* ≤ 0.05.

## SUPPLEMENTARY MATERIALS FIGURES AND TABLES


