# Supplementary material for: KLF4α stimulates breast cancer cell proliferation by acting as a KLF4 antagonist
Source: Oncotarget. 2016 Jun 15;7(29):45608–21. doi: 10.18632/oncotarget.10058 (PMC5216746; doi:10.18632/oncotarget.10058)
Supplement: Supplementary file 1 [file oncotarget-07-45608-s001.pdf]

KLF4α stimulates breast cancer cell proliferation by acting as a KLF4 antagonist

Supplementary Materials

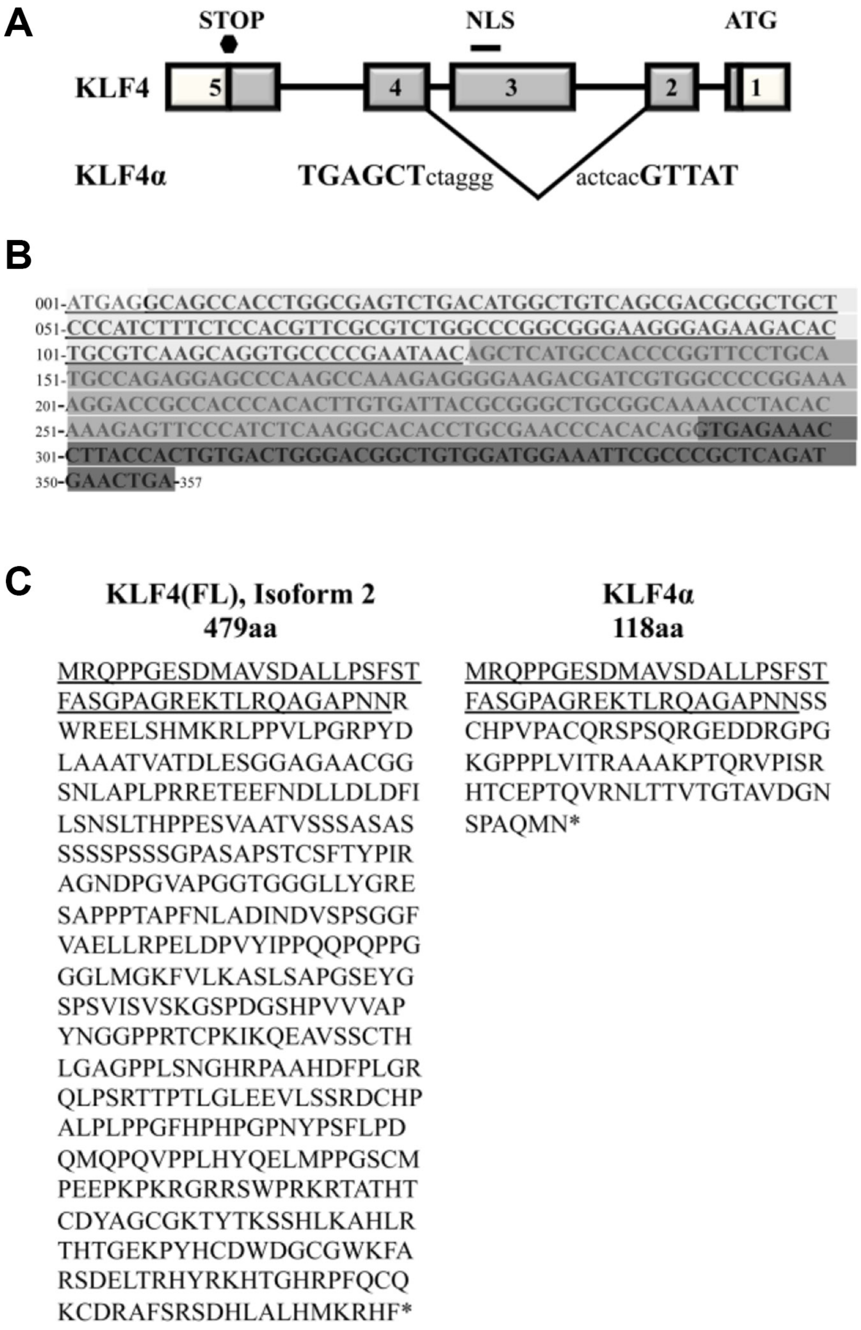

Supplementary Figure S1: cDNA and protein sequence of KLF4α in MDA-MB-231 cells. (**A**) Schematic representation of the *KLF4* gene. Splicing site sequence for *KLF4α* is indicated below. Letters in uppercase: exon part, letters in lowercase: intron part. (**B**) cDNA sequence of KLF4α. The different gray shades indicate the 4 different exons present. (**C**) Comparison of the KLF4(FL) and KLF4α protein sequence. Underlined sequence: 42 aa, which are common between KLF4α and KLF4(FL).

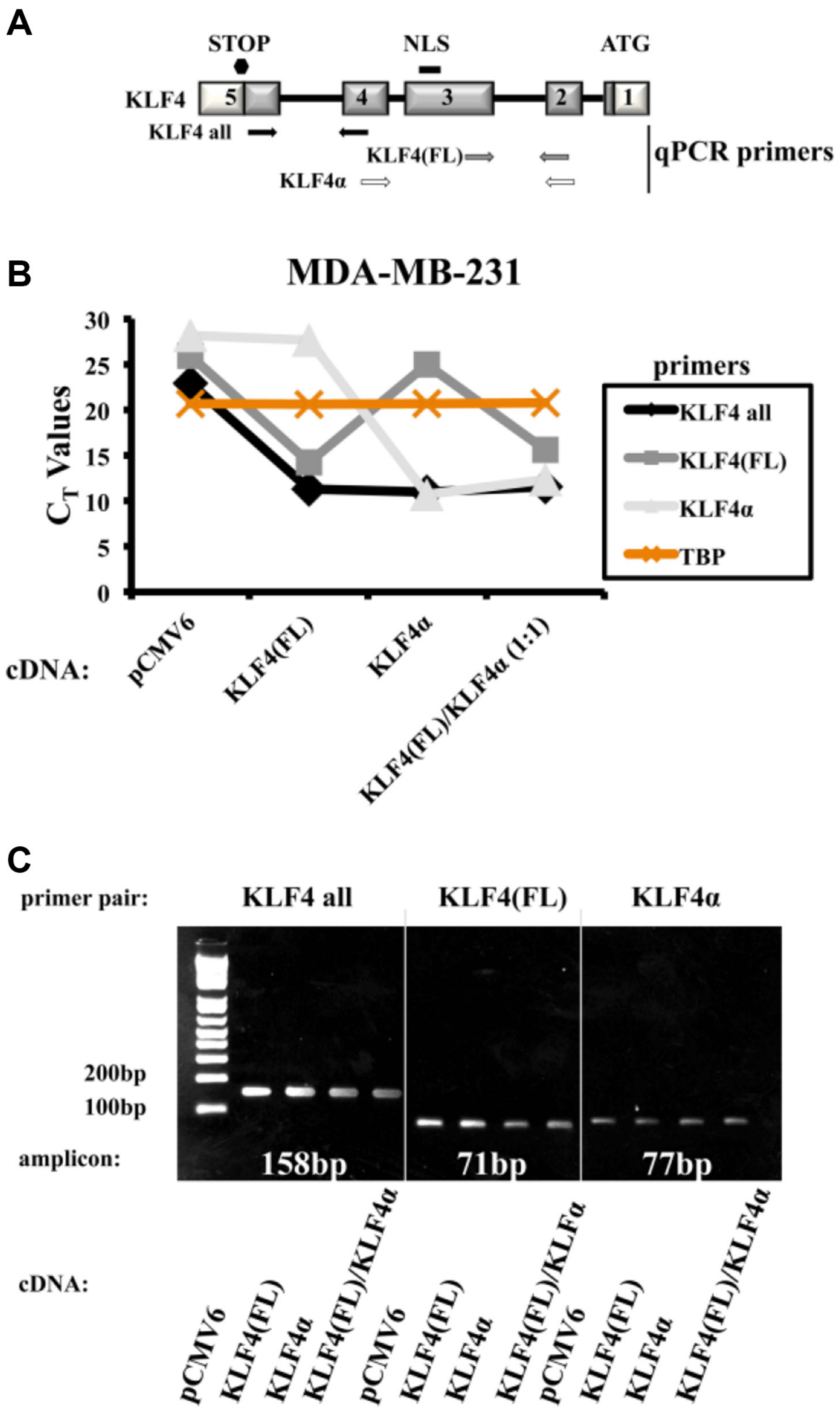

Supplementary Figure S2: KLF4α primer specificity. (**A**) Schematic representation of the *KLF4* gene and the binding sites of the specific primer pairs. (**B**) qPCR analysis of transiently transfected MDA-MB-231 cells with the expression plasmids pCMV6, KLF4(FL), KLF4α, and a 1:1 mix of KLF4(FL) and KLF4α 24 h after transfection using specific primers that recognize all KLF4 isoforms (*KLF4 all*), KLF4(FL) only (*KLF4(FL)*), and KLF4α (*KLF4*α). Note that the primers show the expected specificities. TBP: TATA-Box binding protein. (**C**) qPCR amplicons were analyzed on an agarose gel. For each primer pair, a single band was detectable corresponding to the expected size.

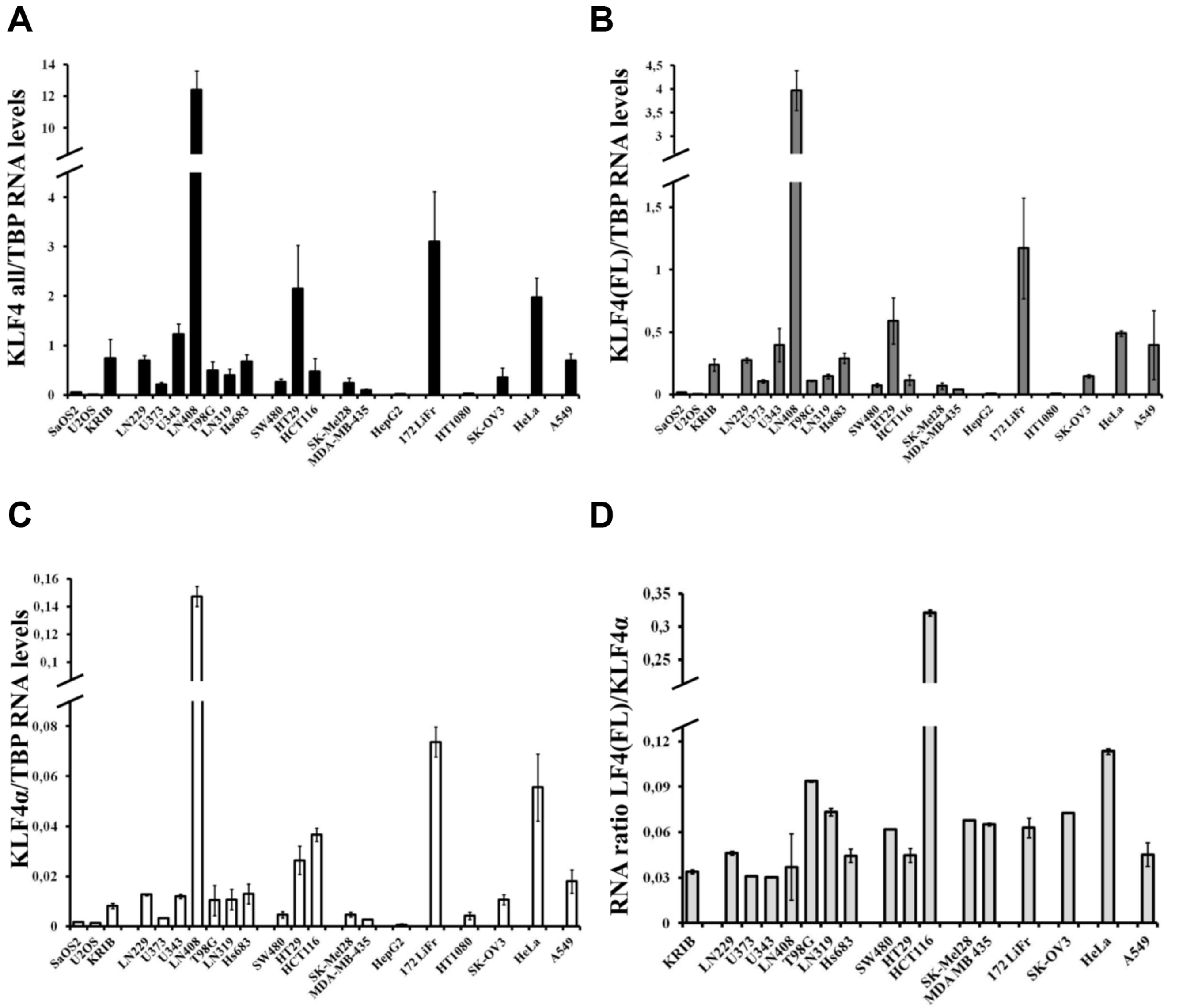

Supplementary Figure S3: *KLF4α* expression in different cancer cell lines. A panel of 21 human cancer cell lines was analyzed by qPCR for *KLF4 all* (top left), *KLF4(FL)* (top right), *KLF4*α (bottom left) and the RNA ratio *KLF4*α*/KLF4(FL)* (bottom right). In most of the cell lines (except for SaOS2, U2OS, HepG2) *KLF4*α was detectable. Levels of *KLF4(FL)* and *KLF4*α, as well as the resulting *KLF4*α*/ KLF4(FL)* ratio vary across the cell lines analyzed. TBP: TATA-Box binding protein.

TissueScan: Clinical data

Breast

ID	gender	age	appearance	diagnosis	stage	normal	tumor	stroma	necrosis
N	F	47	Normal		Normal	100	0	0	0
T1	F	52	Tumor	ductal Adenocarcinoma	IIA	0	70	30	0
T2	n.a.	78	Tumor	ductal Adenocarcinoma	IIA	0	85	10	5
T3	F	50	Tumor	ductal Adenocarcinoma	IIB	0	85	13	2
T4	F	55	Tumor	ductal Adenocarcinoma	IIIC	10	80	10	0
T5	F	68	Tumor	ductal Adenocarcinoma	IIIC	0	90	10	0

Kidney

ID	gender	age	appearance	diagnosis	stage	normal	tumor	stroma	necrosis
N	M	77	Normal		Normal	100	0	0	0
T1	M	43	Tumor	Carcinoma, renal cell, papillary	I	0	75	10	15
T2	M	64	Tumor	Carcinoma, renal cell, papillary	II	0	95	5	0
T3	M	4	Tumor	Nephroblastoma	II	0	95	0	5
T4	M	59	Tumor	Carcinoma, renal cell, papillary	II	0	70	0	30
T5	M	59	Tumor	Carcinoma, renal cell, clear cell	III	0	75	10	15

Lung

ID	gender	age	appearance	diagnosis	stage	normal	tumor	stroma	necrosis
N	F	72	Normal		Normal	100	0	0	0
T1	F	82	Tumor	Adenocarcinoma	IA	0	40	55	5
T2	M	65	Tumor	Squamous cell carcinoma	IIB	10	65	24	1
T3	M	76	Tumor	Squamous cell carcinoma	IIIA	0	60	10	30
T4	F	46	Tumor	Adenocarcinoma	IV	25	40	35	0
T5	F	64	Tumor	carcinoid, atypical	n.a.	0	90	6	4

Ovary

ID	gender	age	appearance	diagnosis	stage	normal	tumor	stroma	necrosis
N	F	54	Normal		Normal	100	0	0	0
T1	F	58	Tumor	Adenocarcinoma, endometrioid	IIB	0	60	15	25
T2	F	91	Tumor	Adenocarcinoma, papillary serous	III	0	90	10	0
T3	F	49	Tumor	Tumor of ovary, mucinous, borderline	IIIA	0	30	70	0
T4	F	47	Tumor	Adenocarcinoma, clear cell	IIIC	0	65	35	0
T5	F	68	Tumor	Adenocarcinoma, serous	IV	10	50	10	30

Supplementary Figure S4: Clinical date of the TissueScans. A TissueScan of breast, kidney, lung, and ovary cancer patients was used to study RNA levels of *KLF4(FL)* and *KLF4α*. Clinical data of the clinical specimens are summarized. Numbers (last four columns) represent percentage of the indicated tissues.

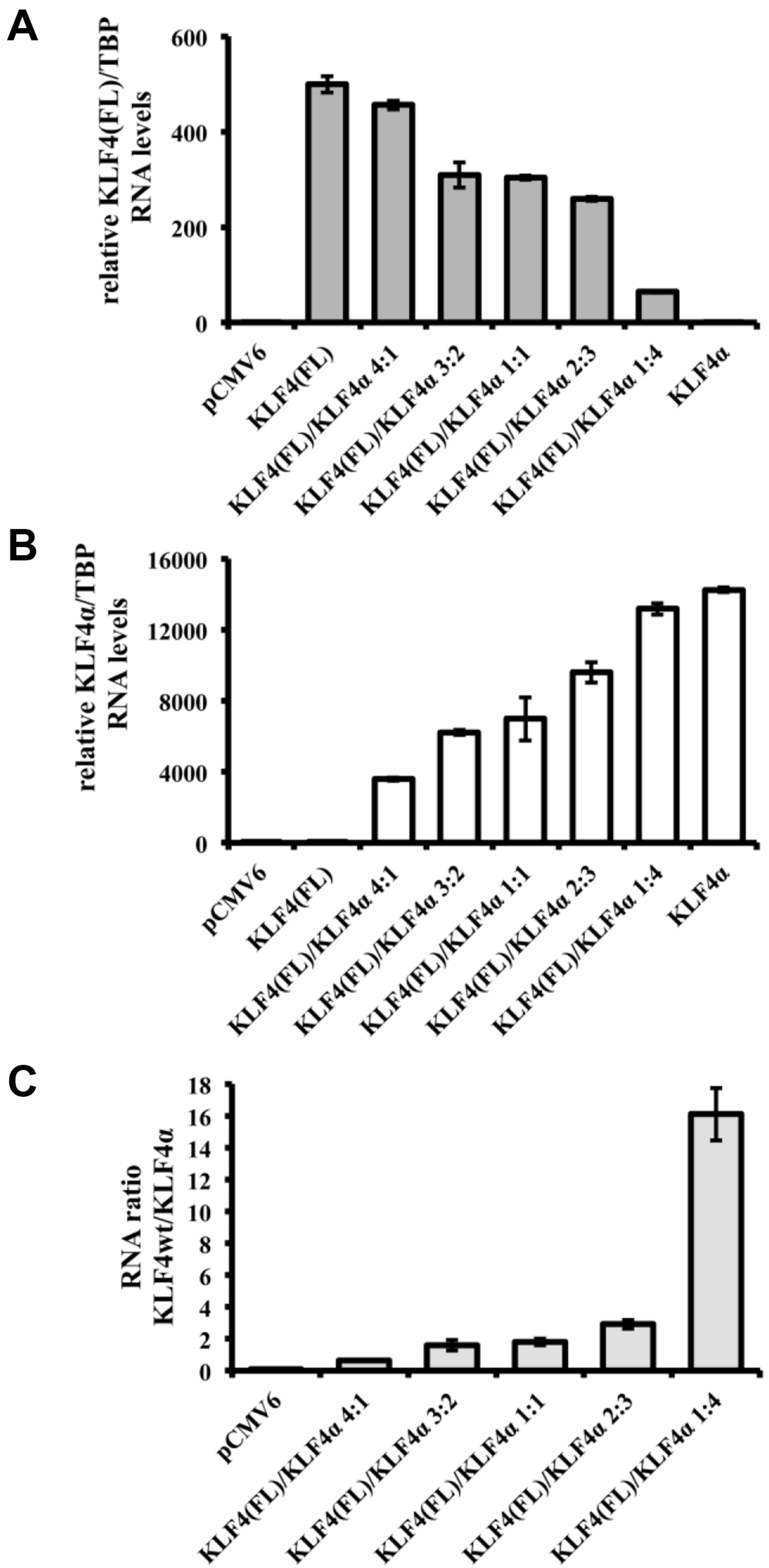

Supplementary Figure S5: *KLF4(FL)* and *KLF4α* levels in transfected MDA-MB-231 cells. (**A**) qPCR analysis of *KLF4(FL)* RNA in MDA-MB-231 cells transfected with different KLF4(FL)/KLF4α ratios. (**B**) qPCR analysis of *KLF4*α levels in MDA-MB-231 cells transfected with different KLF4(FL)/KLF4α ratios. (**C**) Ratio of forced KLF4(FL)/KLF4α imbalance in MDA-MB-231 cells. TBP: TATA-Box binding protein.

Supplementary Table S1: Details of the cell lines used

NAME	ATCC♯	TISSUE	DISEASE	MEDIUM
MCF10A	CRL-10317	Breast		DMEM/F12, Horse serum, cholera toxin, EGF, Hydrocortisone, Insulin
MCF7	HTB-22	Breast	adenocarcinoma; ER+	DMEM
T47D	HTB-133	Breast	ductal carcinoma; ER+, PR+	DMEM
MDA-MB-175	HTB-25	Breast	ductal carcinoma	DMEM/F12
MDA-MB-231	HTB-26	Breast	adenocarcinoma; ER+	DMEM
Saos-2	HTB-85	Bone	osteosarcoma	DMEM
U-2 OS	HTB-96	Bone	osteosarcoma	DMEM
143B (KRIB)	CRL-8303	Bone	osteosarcoma	DMEM
LN229	CRL-2611	Brain	glioblastoma	DMEM
U373	HTB-17	Brain	glioblastoma	DMEM
U343		Brain	glioblastoma	DMEM
LN408		Brain	glioblastoma	DMEM
T98G	CRL-1690	Brain	glioblastoma	DMEM
LN319		Brain	glioblastoma	DMEM/1% glutamine
Hs683	HTB-138	Brain	oligodendroglioma	DMEM/1% glutamine
SW480	CCL-228	Colon	colorectal adenocarcinoma	Leibovitz's L15
HT29	HTB-38	Colon	colorectal adenocarcinoma	McCoy's
HCT116	CCL-247	Colon	colorectal adenocarcinoma	McCoy's
Sk-Mel-28	HTB-72	Skin	malignant melanoma	DMEM
MDA-MB-435	HTB-129	Skin	melanoma	Leibovitz's L15
172			LiFraumeni (Trp53R172H/R172H)	DMEM
Hep G2	HB-8065	Liver	hepatocellular carcinoma	EMEM
HT1080	CCL-121	Connective	fibrosarcoma	DMEM
SK-OV3	HTB-77	Ovary	adenocarcinoma	McCoy's
HeLa	CCL-2	Cervix	adenocarcinoma	DMEM
A549	CCL-185	Lung	carcinoma	RPMI 1640/glutamine

Supplementary Table S2: qPCR primers

gene	Sequence (5′-3′)
KLF4 all F	CAAGCCAAAGAGGGGAAGAC
KLF4 all R	CGTCCCAGTCACAGTGGTAA
KLF4(FL) F	GAGAAGACACTGCGTCAAGC
KLF4(FL) R	AGTCGCTTCATGTGGGAGA
KLF4α F	GTGCCCCGAATAACAGCTCA
KLF4α R	ACGATCGTCTTCCCCTCTTT
$p21^{Cip1}$ *F*	TGGAGACTCTCAGGGTCGAAA
$p21^{Cip1}$ *R*	CGGCGTTTGGAGTGGTAGAA
$p27^{Kip1}$ *F*	CCGGTGGACCACGAAGAGT
$p27^{Kip1}$ *R*	GCTCGCCTCTTCCATGTCTC
CDH1 F	AGAACGCATTGCCACATACACT
CDH1 R	TCTGATCGGTTACCGTGATCAA
TBP F	TGCACAGGAGCCAAGAGTGAA
TBP R	CACATCACAGCTCCCCACCA

F: forward; R: reverse.
